# Protective effects of *Lactococcus lactis* subsp. *lactis* HFY14 supplementation on the brain, intestines, and motor function of antibiotic-treated mice

**DOI:** 10.3389/fmicb.2024.1418556

**Published:** 2024-06-14

**Authors:** Yang Yang, Yuanji Zhao, Huan Lei

**Affiliations:** ^1^College of Physical Education, Chengdu Sport University, Chengdu, Sichuan, China; ^2^School of Physical Education, Wuhan Sports University, Wuhan, Hubei, China

**Keywords:** *Lactococcus lactis* subsp. *lactis*, intestine, sports, antibiotic, ICR mice

## Abstract

**Introduction:**

This study aimed to explore the anti-oxidative and anti-inflammatory properties of *Lactococcus lactis subsp*. *lactis* HFY14 (LLSLHFY14) and investigate its effects on the intestinal barrier, cranial nerve, and motor function in mice treated with antibiotics.

**Methods:**

Mice were administered an antibiotic mixture (neomycin 5 mg/mL, vancomycin 25 mg/mL, amphotericin B 0.1 mg/mL, ampicillin 10 mg/mL, metronidazole file 5 mg/mL, and lipopolysaccharide 1.5 μg/mL) intraperitoneally, and oxidative stress and inflammatory markers in the serum and brain tissues, and liver index were measured. H&E staining was performed to detect pathological alterations in brain tissues. The expression of intestinal-barrier-related genes and that of genes involved in inflammatory pathways in the brain were detected using polymerase chain reaction (PCR).

**Results:**

LLSLHFY14 administration extended the weight-loaded swimming and running times of mice and decreased the liver index. Moreover, the levels of malondialdehyde (MDA), interleukin-6 (IL-6), and tumor necrosis factor alpha (TNF-α) in the serum and brain tissue were reduced, whereas those of superoxide dismutase (SOD), glutathione (GSH), and interleukin-10 (IL-10) were elevated. Elevated brain expression of the protein kinase B (*AKT*)/cAMP-response element binding protein (*CREB*)/brain-derived neurotrophic factor (*BDNF*)/extracellular signal-regulated kinase 1 (*ERK1*) pathway, decreased brain expression of the *IL-6* gene, and elevated cecum expression of *zonula occludens-1 (ZO-1), occludin-1*, and *claudin-1* genes were noted. LLSLHFY14 supplementation significantly increased *Bacteroidetes* expression but decreased *Firmicutes* expression, thus increasing the *Bacteroidetes/Firmicutes* ratio.

**Discussion:**

Overall, LLSLHFY14 supplementation ameliorated antibiotic-induced oxidative stress and inflammation in the mouse central nervous system, intestinal barrier dysfunction, and increased motor function, thus confirming its potential application as probiotics.

## 1 Introduction

Antibiotics are frequently used in the treatment of human infections owing to their bactericidal properties. However, antibiotic misuse has been linked to adverse health effects in humans, such as allergies, renal toxicity, obesity, gut dysbiosis, depression, and brain neuropathies and is a public health issue, as it causes bacterial resistance (Wright, 2005; Hemamalini et al., 2021). Dysbiosis of the gut microbiota due to antibiotic overuse results in several intestinal disorders and central nervous system dysfunction and increases the permeability of the blood–brain and intestinal barriers. When these barriers are broken, bacteria and their metabolites enter the bloodstream, inducing damage to the central nervous system (Wright, 2005; Yang et al., 2023). Moreover, the occurrence and development of diabetes, anxiety, fatty liver disease, and Alzheimer’s disease are strongly correlated with the composition of the gut flora (Zhang et al., 2022). Thus, a well-balanced gut microbiota is important to maintain normal physiological functions, such as digestion, immunity, and metabolism. Recently, there has been increasing research attention on the “microbiota–gut–brain axis” and the use of gut microbiota as a therapeutic target improving physical fitness and preventing diseases (Lyte, 2014; Zhu et al., 2017).

Probiotic supplementation can ameliorate various adverse responses induced by antibiotic misuse. *Lactobacillus*, *Bacillus*, *Bifidobacterium, Bacillus subtilis*, and some streptococcal probiotics are current research hotspots, with the primary products being food, pharmaceuticals, and healthcare products (Hummel et al., 2007). Probiotics work primarily by maintaining the integrity of the intestinal barrier, inhibiting the adherence of pathogenic bacteria, and probiotics can decompose dietary fiber to produce short chain fatty acids and other nutrients, which are easily absorbed by the intestine to obtain energy and nutrients, enhance the immune system to enhance immune function, and reduce the incidence of inflammation. Probiotics can enhance human immunity and regulate the gut microbiota composition and function by secreting nutrients, enzymes, and secondary metabolites that have health-promoting properties. This can directly or indirectly improve the physiological functions of the gut and the central nervous (Ng et al., 2019).

*Lactococcus lactis* subsp. *lactis* HFY14 is a lactic acid bacteria isolated from naturally fermented yak yogurt on the Qinghai Tibet Plateau in China. Due to the unique geographical environment of the Qinghai Tibet Plateau and the lifestyle habits of Tibetan herdsmen, the microorganisms in yak yogurt consumed have good resistance. The *in vitro* activity of LLSLHFY14 against artificial gastric acid and 3% artificial bile salt reached 84.26% and 54.31%, respectively. Therefore, LLSLHFY14 with good resistance was selected for further research in this study. In this study, antibiotic-induced damage and microbiota disturbance were evaluated to determine whether LLSLHFY14 intervention has protective effects on motor function, intestinal permeability, and brain inflammation and oxidative stress in mice.

## 2 Materials and methods

### 2.1 Cultivation of experimental strains

The *Lactococcus lactis* subsp. *lactis* HFY14 strain (preservation number: 18227), which was isolated and identified from naturally fermented yogurt in Hongyuan County, Sichuan Province, China was obtained from the Chinese General Microbiological Culture Collection Center (No. 16647, Beijing, China). The strain was stored at −80°C, thawed, inoculated (200 μl) into 8 ml of Man Rogosa Sharpe (MRS; Solarbio, Beijing, China) liquid medium, and cultivated for 24 h at 37°C. Then, 3% of the inoculum was mixed in and left on MRS liquid medium for another 18 h. Then, it was centrifuged at 4°C and 4,000 rpm for 10 min. The supernatant was collected and resuspended in normal saline solution, and the cell concentration was adjusted to approximately 1.0 × 10^9^ CFU/kg ⋅ bw.

### 2.2 Animal models

Institute of Cancer Research (ICR) mice (7 weeks old, male, Hunan Slyke Jingda Laboratory Animal Co., Ltd., Changsha, Hunan, China) were kept in normal cages at 23 ± 2°C with a relative humidity of 55% ± 5% and acclimatized for a week. They were given unlimited access to food and water at 12/12 h dark/light conditions and were randomly and equally divided into the following groups: normal, model, caffeic acid [CA, Aladdin Reagent (Shanghai) Co., Ltd., Shanghai, China], LLSLHFY14 low concentration, and LLSLHFY14 high concentration, with 10 mice in each group. Group 1 mice (normal) received no intervention; group 2 (model), intraperitoneal injection of the antibiotic mixture and intragastric administration of normal saline; group 3 (CA), intraperitoneal injection of the antibiotic mixture and gavage of CA (10 mg/kg); group 4 (LLSLHFY14LD), intraperitoneal injection of low concentration of LLSLHFY14 (10^8^ CFU/kg of body weight); and group 5 (LLSLHFY14HD), intraperitoneal injection of the antibiotic mixture and gavage of high concentration of LLSLHFY14 (10^9^ CFU/kg of body weight). Experimental treatment was performed for 2 weeks. The antibiotic mixture comprised 5 mg/ml of neomycin, 25 mg/ml of vancomycin, 0.1 mg/ml of amphotericin B, 10 mg/ml of ampicillin, 5 mg/ml of metronidazole [Aladdin Reagent (Shanghai) Co., Ltd.], and 1.5 μg/ml of lipopolysaccharide (Desbonnet et al., 2015).

### 2.3 Weight-loaded forced swimming test

Mice were placed in a tank filled with water to a depth of 35 cm and maintained at 20 ± 2°C. The tank dimensions were Φ 60 cm × 5 cm. They were marked and carrying 5% of their body weight attached to their tails. After 30 min of dosing, mice were submerged in water. They were considered exhausted with failure to rise above water within 8 s. After the test, mice were patted dry using towels.

### 2.4 Running platform experiment

An experimental mice running platform for ZH-PT (Anhui Zhenghua Biological Instrument Equipment Co., Ltd., Huaibei, Anhui, China) was used measuring 565 mm × 630 mm × 310 mm in length, width, and height, respectively. It has eight running platforms and a single track of 55 mm. Test conditions were as follows: tilt angle, 5°; stimulating current, 0.6 mA; acceleration, 2 m/s^2^; and speed, 20 m/min. The exercise period lasted 30 min, and the rest time was 1 min. Four days before to the experiment’s conclusion, adaptive training was initiated once daily for 5 min. The training was officially tested on the last day of the trial, and outcomes were noted.

### 2.5 Tissue collection

After administration of ether anesthesia to mice, blood was extracted from their eyes, and mice were weighed after the exercise trial. After the mice were euthanized, their brain (frontal lobe), liver, and cecum tissues were collected. Liver tissues were weighed. A portion of the brain tissue was immersed in tissue fixative. The remaining tissue was stored at −80°C for future analysis.

### 2.6 Pathological observation

After fixing in a solution for more than 48 h, brain frontal lobe tissues (0.3 cm^2^) were paraffinized, sectioned (4 μm), stained with hematoxylin and eosin, and visualized under an electron microscope to evaluate pathological changes (BX43 light microscope, Olympus, Tokyo, Japan).

### 2.7 Determination of serum and brain tissue biochemical indicators

Mouse blood samples were kept standing at 4°C for 2 h, centrifuged at 3,000 rpm for 15 min at 4°C, and the upper layer was collected and stored frozen at −80°C. Mouse brain tissue (0.1 g) was homogenized thrice for 30 s at 6 m/s and added with 0.9 ml of normal saline. The levels of superoxide dismutase (SOD), glutathione (GSH), and malondialdehyde (MDA) in mouse brain homogenate and serum were measured using their respective kits (Solarbio). The levels of tumor necrosis factor alpha (TNF-α), interleukin (IL)-6, and IL-10 in the serum and brain tissue were determined using enzyme-linked immunosorbent assay kits (Shanghai Enzyme linked Biotechnology Co., Ltd., Shanghai, China).

### 2.8 Real-time fluorescence quantitative PCR

Following the homogenization of brain and cecum tissues, TRIzol reagent (Solarbio) was used to extract total RNA, and the concentration was diluted to 1.0 μg/μl. cDNA was synthesized after reverse transcription of total RNA. The reaction mixture contained 1.0 μl of cDNA, 2.0 μl each of the forward and reverse primers (10 μm) (Thermo Fisher Scientific, Waltham, MA, USA), and 10 μl of the premix. The thermal cycling conditions were as follows: 40 cycles for 30 s at 60°C, 1 min at 95°C, and 3 min at 95°C (StoponePlus, Thermo Fisher Scientific). For quantitative analysis, β-actin was used as the reference gene for normalization, and the 2^–ΔΔCt^ method was used (Zhao et al., 2019).

### 2.9 Determination of microbial mRNA expression in mouse feces

Microbial mRNA expression in mouse fecal samples (1.0 g) was measured using the tissue mRNA assay to detect the microbial composition in the feces.

### 2.10 Statistical analysis

Statistical analysis was performed using the Tukey technique for multiple comparisons and one-way analysis of variance in SPSS version 20.0 (SPSS Inc., Chicago, IL, USA). Statistical significance was set at *p* < 0.05. To plan, Microsoft Excel version 2019 was used. Data were replicated thrice and are presented as the mean ± standard deviation (SD).

## 3 Results

### 3.1 Weight-loaded forced swimming test

**FIGURE 1:**
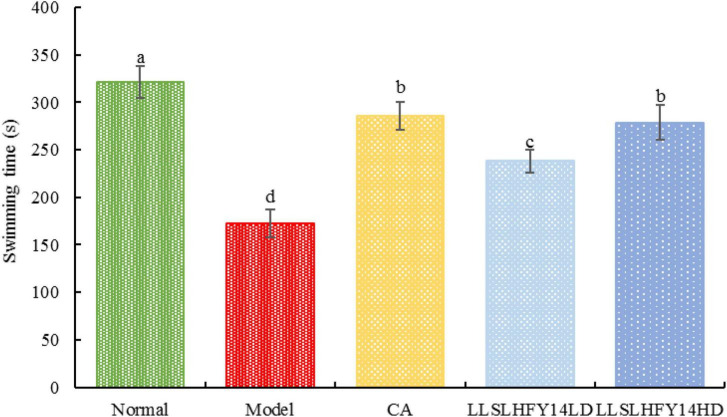
Changes in weight-bearing swimming time in antibiotic-treated mice. ^a–d^Values presented are the mean ± standard deviation, mean values with different letters in the same graph are significantly different (*p* < 0.05) according to Tukey’s test, the same below.

Mouse endurance may be reduced by antibiotic use, but it may be improved by LLSLHFY14 intervention. The swimming time of the model group was considerably decreased (*p* < 0.05) than that of the normal group (Figure 1). The swimming time rose dramatically in the gavage intervention group of LLSLHFY14, approaching that in the CA and normal groups. There was a significant (*p* < 0.05) between the LLSLHFY14LD and LLSLHFY14HD groups.

### 3.2 Running experiment

**FIGURE 2:**
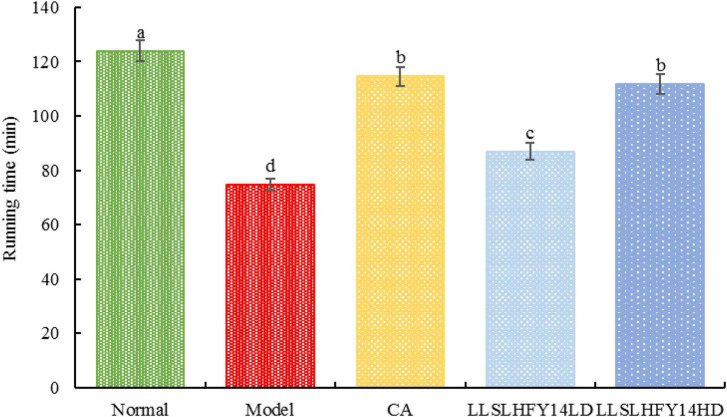
Changes in running time in antibiotic-treated mice.

As shown in Figure 2, the model group’s running time was considerably (*p* < 0.05) shorter than the normal group’s running time. Running duration increased dramatically in the group with early gavage intervention with LLSLHFY14 and caffeic acid, with the LLSLHFY14HD group showing superior results than the LLSLHFY14LD group. The running time of the model group was much lower than that of the CA group but was significantly higher than that of the LLSLHFY14 and LLSLHFY14HD groups. The CA group’s running time was shorter than the normal group’s.

### 3.3 Changes in mouse liver index

The model group’s liver index was significantly higher than the normal group’s (Table 1; *p* < 0.05). Following the intervention of LLSLHFY14, the liver index decreased, with that of the LLSLHFY14HD group being statistically significant and resembling that of the CA group.

**TABLE 1:** Body weight, liver weight, and liver index of antibiotic-treated mice.

Group	Body weight (g)	Liver weight (g)	Liver index
Normal	31.18 ± 3.28^a^	0.86 ± 0.07^a^	2.76 ± 0.29^b^
Model	23.77 ± 2.12^c^	0.91 ± 0.04^a^	3.88 ± 0.48^a^
CA	27.79 ± 2.86^ab^	0.85 ± 0.06^a^	3.07 ± 0.37^ab^
LLSLHFY14LD	25.52 ± 2.36^b^	0.85 ± 0.05^a^	3.35 ± 0.43^ab^
LLSLHFY14HD	27.28 ± 1.86^ab^	0.83 ± 0.05^a^	3.07 ± 0.27^ab^

^a–d^Values presented are the mean ± standard deviation, mean values with different letters in the same column are significantly different (*p* < 0.05) according to Tukey’s test, the same below.

### 3.4 Pathological changes in mouse brain tissues

**FIGURE 3:**
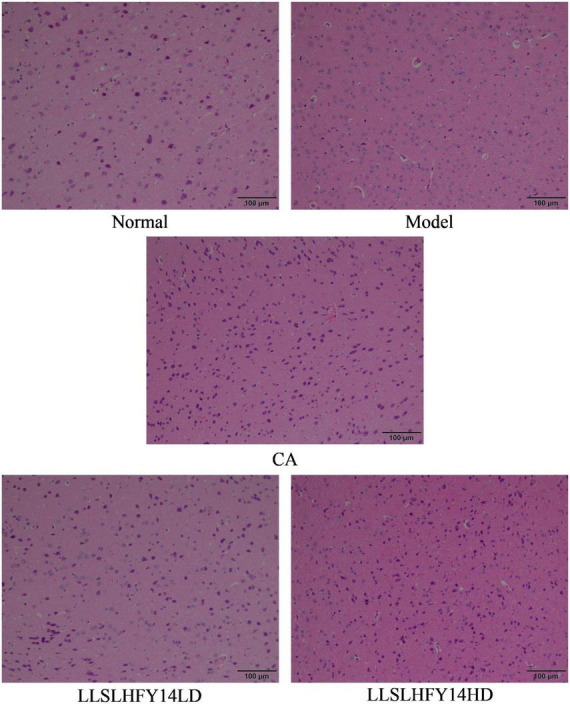
Pathological changes of brain tissues in antibiotic-treated mice (40×).

Pathological observation can preliminarily qualitatively observe the degree of visceral damage in experimental animals. Figure 3 shows that the neurons in the cerebral cortex of mice in the normal group had normal morphology, uniform, well-arranged cells, and prominent nuclei. Within the scope of the microscope’s field of view, the model group had a reduced number of cells, dark nuclei color, evidence of pyknosis, punctate necrosis, and uneven arrangement. Cells showed severe damage, and the cortical neurons were smaller in size. Following pretreatment with LLSLHFY14, the brain tissues showed less karyopyknosis and an increased number of cells. Although some neurons shrank in size and showed signs of atrophies, most neurons had a normal appearance. The changes in the CA and LLSLHFY14HD groups were similar.

### 3.5 Serum oxidative and inflammatory markers

The serum levels of MDA were significantly higher in the model group than in the normal group, whereas those of GSH and CuZn-SOD were significantly lower (Table 2; *p* < 0.05). Compared to that of the model group, following CA, LLSLHFY14LD, and LLSLHFY14HD pretreatment, the MDA serum level decreased, but those of GSH and CuZn-SOD increased; these differences were statistically significant (*p* < 0.05). The model group had higher levels of pro-inflammatory factors TNF-α and IL-6 and lower levels of IL-10, an anti-inflammatory factor, than the normal group (Table 3; *p* < 0.05). Compared to those of the model group, pretreatment with CA, LLSLHFY14LD, and LLSLHFY14HD significantly reduced the TNF-α and IL-6 levels but increased IL-10 levels (*p* < 0.05). The effects in the CA and LLSLHFY14HD groups were comparable.

**TABLE 2:** Oxidation and inflammation indicators of serum in antibiotic-treated mice.

Group	GSH (μmol/L)	CuZn-SOD (U/ml)	MDA (nmol/ml)	IL-10 (pg/ml)	IL-6 (pg/ml)	TNF-α (pg/ml)
Normal	140.83 ± 11.36^a^	173.08 ± 21.10^a^	4.86 ± 0.62^c^	25.41 ± 3.44^a^	33.90 ± 1.39^d^	61.85 ± 7.00^d^
Model	64.37 ± 9.29^c^	65.57 ± 21.14^c^	11.12 ± 1.31^a^	3.45 ± 2.45^c^	55.92 ± 2.12^a^	139.45 ± 8.24^a^
CA	129.57 ± 10.36^a^	131.51 ± 25.91^ab^	6.75 ± 1.10^b^	17.02 ± 5.18^ab^	41.50 ± 1.87^c^	81.61 ± 7.35^c^
LLSLHFY14LD	88.26 ± 11.01^b^	79.46 ± 23.57^c^	8.98 ± 1.24^b^	6.13 ± 2.72^c^	47.93 ± 2.05^b^	112.47 ± 8.79^b^
LLSLHFY14HD	124.03 ± 9.65^a^	121.41 ± 29.45^b^	7.05 ± 1.27^b^	15.23 ± 3.38^b^	42.09 ± 1.81^c^	83.77 ± 6.37^c^

^a–d^Values presented are the mean ± standard deviation, mean values with different letters in the same column are significantly different (*p* < 0.05) according to Tukey’s test, the same below.

**TABLE 3:** Oxidation and inflammation indicators of brain tissue in antibiotic-treated mice.

Group	GSH (μmol/mg prot)	CuZn-SOD (U/mg prot)	MDA (nmol/mg prot)	IL-10 (pg/mg prot)	IL-6 (pg/mg prot)	TNF-α (pg/mg prot)
Normal	17.18 ± 0.54^a^	47.37 ± 0.40^a^	3.56 ± 0.78^d^	32.96 ± 1.15^a^	7.53 ± 0.55^d^	119.90 ± 13.27^d^
Model	7.84 ± 0.29^d^	25.83 ± 0.32^d^	14.21 ± 1.10^a^	17.13 ± 0.81^d^	19.62 ± 0.81^a^	262.91 ± 12.84^a^
CA	14.86 ± 0.60^b^	39.47 ± 0.37^b^	5.27 ± 0.43^c^	27.39 ± 0.95^b^	9.30 ± 0.22^c^	168.54 ± 14.86^c^
LLSLHFY14LD	11.58 ± 0.61^c^	35.38 ± 0.44^c^	9.16 ± 0.67^b^	20.62 ± 0.27^c^	13.04 ± 0.72^b^	203.18 ± 13.36^b^
LLSLHFY14HD	14.32 ± 0.69^b^	39.32 ± 0.57^b^	5.87 ± 0.39^c^	26.49 ± 1.27^b^	9.41 ± 0.51^c^	174.36 ± 8.60^c^

^a–d^Values presented are the mean ± standard deviation, mean values with different letters in the same column are significantly different (*p* < 0.05) according to Tukey’s test, the same below.

### 3.6 Indexes of oxidation and inflammation in mouse brain tissue

The model group’s brain tissue had significantly higher MDA levels and lower GSH and CuZn-SOD levels than the normal group’s brain tissue (Table 2; *p* < 0.05). Following LLSLHFY14 intervention, the GSH and CuZn-SOD levels increased, compared to those in the model group, with nonsignificant increases between the LLSLHFY14HD and CA groups. Better than LLSLHFY14LD, CA, and LLSLHFY14HD both reduced the MDA level, with comparable effects. The model group had higher levels of TNF-α and IL-6 but lower levels of IL-10 than the normal group (Table 3; *p* < 0.05). Pretreatment with CA and LLSLHFY14 increased IL-10 levels but decreased TNF-α and IL-6 levels (CA > LLSLHFY14HD > LLSLHFY14LD). These three effects also showed opposing tendencies.

### 3.7 Gene expression of *AKT*, *BDNF*, *CREB*, and *ERK1* in mouse brain tissue

**FIGURE 4:**
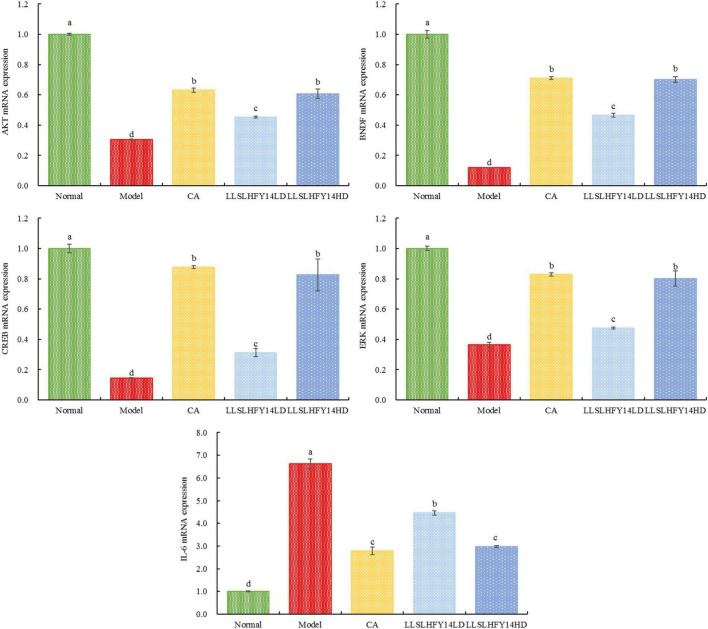
mRNA expression of *AKT, BDNF, CREB*, *ERK1*, and *IL-6* in mouse brain tissue.

Significant roles for protein kinase B (*AKT*), cAMP-response element binding protein (*CREB*), brain-derived neurotrophic factor (*BDNF*), extracellular signal-regulated kinase 1 (*ERK1*), and interleukin-6 (*IL-6*) are seen in cranial nerve damage. Figure 4 shows the real-time fluorescence quantitative polymerase chain reaction (PCR) results. The expression of the *AKT, CREB, BDNF*, and *ERK1* genes was significantly lower (*p* < 0.05) in the model group than in the normal group. Their expression increased in the CA and LLSLHFY14HD groups. The gene expression increase was higher in the LLSLHFY14LD group than in the LLSLHFY14HD group. Gene expression in the LLSLHFY14HD groups was comparable to that in the normal and CA groups. The model group showed the strongest expression of *IL-6*, LLSLHFY14HD and CA groups are close to the normal group.

### 3.8 Gene expression of *ZO-1*, *occludin-1*, and *claudin-1* in mouse cecum

**FIGURE 5:**
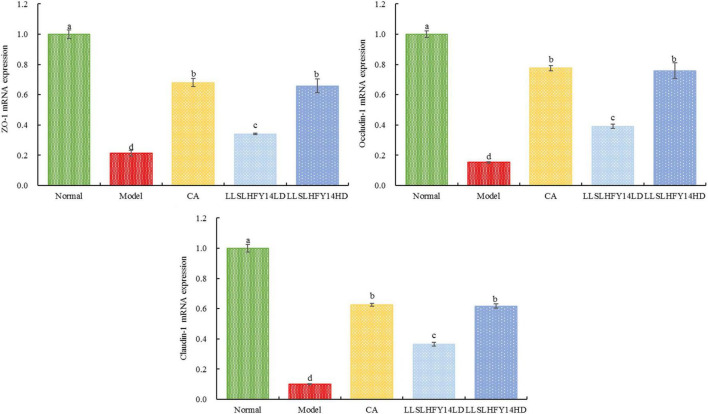
mRNA expression of *ZO-1, occludin-1*, and *claudin-1* in mouse cecum.

*ZO-1*, *occludin-1*, and *claudin-1* are traditional tight junction (TJ) proteins and can indicate the degree of structural damage in the TJ and the mechanical barrier function in intestinal epithelial cells. The model group had lower expression of *ZO-1, occludin-1*, and *claudin-1* genes than the normal group (Figure 5; *p* < 0.05). Following CA and LLSLHFY14 intervention, their expression increased, with the increase being comparable to that in the normal group.

### 3.9 *Firmicutes* and *Bacteroidetes* expression in mouse fecal samples

**FIGURE 6:**
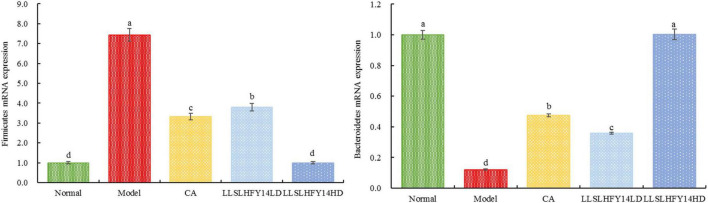
mRNA expression of *Firmicutes* and *Bacteroidetes* in mouse fecal.

As shown in Figure 6, the mRNA abundance of *Firmicutes* was significantly lower in the normal group (*p* < 0.05) than in the other groups, but that of *Bacteroidetes* was significantly higher (*p* < 0.05). The model group showed the lowest *Bacteroidetes* expression and highest *Firmicutes* expression. In mice with thrombosis, the LLSLHFY14 and CA treatments decreased *Firmicutes* expression and increased *Bacteroidetes* expression, showing an enhanced B/F ratio. After two dosages, the changes in gene expression were more pronounced in the LLSLHFY14 group than in the CA group. The changes in the expression of *Firmicutes* and *Bacteroidetes* were normalized by LLSLHFY14HD intervention, showing no discernible change compared to the normal group.

## 4 Discussion

Caffeic acid is a powerful antioxidant that can help eliminate free radicals in the body, delay the aging process, and enhance immunity. Caffeic acid has a stimulating effect on the central nervous system, can refresh the mind, accelerate metabolism, enhance physical fitness, and improve motor function to a certain extent (Girsang et al., 2020). Therefore, in this study, it was selected as a positive control. Probiotics can play a role in intestinal barrier regulation, lower oxidative stress, enhance exercise capacity, provide essential metabolites to skeletal muscle mitochondria, and control the activation of important transcription factors and enzymes, like AMPK and PGC-1α, which are involved in metabolic processes in the mitochondria (Guo et al., 2018; Liu et al., 2021). Probiotic supplements have been demonstrated to enhance lipid metabolism and decrease weight loss and fat accumulation (Ameneh et al., 2016). In this study, mice exhibited longer weight-bearing swimming and running times and increased endurance following LLSLHFY14 intervention. Moreover, LLSLHFY14 intervention resulted in a substantial decrease in the liver index.

Overuse of antibiotics and the combination of multiple antibiotics that are not symptomatic are both manifestations of antibiotic abuse. Therefore, in this study, multiple antibiotics were overmixed to simulate the special situation of antibiotic abuse in clinical practice. This situation is different from the normal use of antibiotics to treat diseases in clinical practice. Neomycin is an antibiotic with a certain degree of nephrotoxicity, and due to its ototoxicity, it may cause gait stumbling, dizziness, or unstable walking, affecting movement. Vancomycin also has nephrotoxicity and may cause muscle spasms. Amphotericin B is highly toxic and has effects on both the liver and kidneys. It can also cause lower limb pain and heart rate imbalance. Excessive use of ampicillin can cause neurological symptoms such as seizures and enteritis. Excessive use of metronidazole can cause liver and kidney dysfunction and seizures. Excessive use of these five antibiotics has significant side effects on metabolism and nerves in the body. In this study, a mixture of these five antibiotics was used to induce visceral dysfunction and motor nerve dysfunction in mice, leading to a decrease in motor ability.

Antibiotic misuse frequently results in a range of disorders that involve oxidative changes to important physiological molecules, such as proteins, lipids, carbohydrates, and nucleic acids, as well as the control of gene expression and the inflammatory response (Kardas et al., 2005). One of the adverse consequences of antibiotic misuse is oxidative damage. The last metabolic byproduct of membrane lipid peroxidation *in vivo* is malondialdehyde (MDA). When MDA is released from the cells, it can alter the permeability of the membrane, resulting in structural and functional damage, and can react with proteins and nucleic acids to initiate cross-linking polymerization and disrupt regular metabolic reactions (Tsikas, 2017). Oxidative stress damage and antioxidant capacity can be significantly improved by endogenous antioxidant enzymes SOD and non-enzymatic GSH (Huang et al., 2022). Our results showed that supplementation of heat-treated or *Lactobacillus rhamnosus* can lower the levels of MDA in both the serum and brain tissues, increase those of SOD and GSH, and limit the degree of peroxidation.

Inflammation is one of the mechanisms by which antibiotics cause brain damage. The dynamic balance between pro-inflammatory and anti-inflammatory responses can be disrupted by exposure to antibiotics and their secondary metabolites, which can increase the levels of inflammatory markers (Bueter et al., 2009). In this study, damaged brain cells and tissue showed pathological alterations. In the cortical area of mice, pyknosis, darker nuclei color, and unorganized arrangement of neurons were noted. It can also enhance the release of pro-inflammatory cytokines, such as TNF-α and IL-6, which can exacerbate brain inflammation and neurotoxicity by activating the immune system. Furthermore, an excessive amount of lipid peroxide and free radicals are generated (Singh and Vinayak, 2015). In this study, LLSLHFY14 supplementation increased the levels of the anti-inflammatory factor IL-10, thereby limiting the degree of inflammation, but decreased those of pro-inflammatory factors TNF-α and IL-6 in both the serum and brain tissue.

Intestinal epithelial cells, the primary constituent of the intestinal barrier system, allow ions, water, and nutrients to enter the body to support metabolism while barring the entrance of dangerous chemicals and infectious agents. Antibiotic exposure changes the shape of the TJs in intestinal epithelial cells, leading to impaired barrier performance. The creation of TJs between adjacent cells is a crucial indicator of the polarity of epithelial cells and intercellular adhesion. The aforementioned activities are based on the high concentration of TJ proteins (Nighot et al., 2015). Claudin-1, occludin, and ZO-1 are examples of TJ proteins in intestinal epithelial cells. They can strengthen the intestinal barrier, prevent the translocation of gut microbiota and enterotoxins to the intestinal mucosa, and improve intestinal inflammation (Inai et al., 2008). Occludin can enhance transmembrane electrical resistance, preserve the structure of intestinal epithelial cells and TJs, and maintain intercellular permeability within a normal range (Liu et al., 2019). Claudin regulates the movement of materials in the intestinal epithelium, and its increased levels cause intestinal epithelial cells to contract (Lei et al., 2012). ZO-1 mediates the opening and closing of TJs by interacting with the actin cytoskeleton across various cells (Tian et al., 2016). This study showed that LLSLHFY14 intervention can improve intestinal barrier damage by increasing the gene expression of *claudin-1, occludin-1*, and *ZO-1* in the gut.

Neurological diseases in the brain are largely influenced by the AKT/CREB/BDNF signaling pathway, which is involved in protein kinase B/CAMP response element binding protein/brain-derived neurotrophic factor (Niu et al., 2022). AKT plays a significant role in the AKT/CREB/BDNF signaling system by regulating cell migration and movement, apoptosis, cell growth and proliferation, and intracellular metabolism (Yan et al., 2016). As a substrate of AKT, CREB controls the transcription of target genes linked to synaptic plasticity, neuronal development, damage, regeneration, and learning and memory capacity (Qu et al., 2015). CREB also enhances the transcription of the CRE sequence. One of the downstream targets of CREB transcription is BDNF, which is controlled by p-CREB since its promoter region contains the CRE sequence (Neal and Guilarte, 2010). When BDNF attaches to its receptor tyrosine kinase B (TrKB), the downstream Ras-Raf-MEK-ERK cascade is triggered. Transcription and translation can be initiated by activation of ERK1/2 to phosphorylate kinases, such as Raf and MEK, which subsequently activate substrates such as CREB to form new synapses (He et al., 2008; Lee et al., 2015). Our findings showed that, following LLSLHFY14 intervention, the expression of *AKT, CREB, BDNF*, and *ERK1* genes in the brain was increased, whereas the levels of the pro-inflammatory factor IL-6 were decreased, suggesting that phosphorylated AKT may activate CREB, increase the expression of BDNF and ERK1, and prevent brain cell damage.

Aberrant lesions in the body often result in ecological dysbiosis, defined as a change in the B/F ratio. Antibiotics significantly affect *Firmicutes* abundance and the production of their metabolites, particularly SCFAs, in the mouse digestive tract (Hu et al., 2020). Alzheimer’s disease is associated with a decreased proportion of *Bacteroidetes*, decreased B/F ratio, and increased proportion of *Firmicutes* (Askarova et al., 2020). These alterations can be ameliorated. In this study, LLSLHFY14 supplementation increased the B/F ratio by lowering *Firmicutes* abundance and increasing *Bacteroidete*s abundance, affecting animal behavior.

## 5 Conclusion

In summary, in this study, the LLSLHFY14 intervention enhanced mouse endurance and increased their swimming and running times until exhaustion. LLSLHFY14 intervention reduced the oxidative and inflammatory reactions induced by antibiotics and regulated the levels of anti-inflammatory and antioxidant markers in both the serum and brain. In addition, LLSLHFY14 can enhance the expression of genes involved in the AKT/CREB/BDNF/ERK1 pathway, which contributes to central neuroprotection in the brain. LLSLHFY14 also reduced the damage to the intestinal epithelial barrier and increased the expression of related intestinal genes in the cecum. By affecting the intestinal microbiota, LLSLHFY14 can also affect the *B/F* ratio in the feces, which subsequently regulates the effect of exercise intervention. Our findings indicate the application of LLSLHFY14 as probiotics to improve oxidative inflammation, intestinal barrier damage, and decreased motor function induced by antibiotics in the central nervous system of mice.

## Data availability statement

The original contributions presented in this study are included in this article/Supplementary material, further inquiries can be directed to the corresponding author.

## Ethics statement

All the experiments were conducted in accordance with the 2010/63/EU directive and the guidelines for the ethical review of laboratory animal welfare of the People’s Republic of China (GB/T 35892-2018) and institutional rules considering animal experiments. Animal experiments have passed the ethical review of experimental animals at Chengdu Sports University. The study was conducted in accordance with the local legislation and institutional requirements.

## Author contributions

YY: Data curation, Writing – original draft. YZ: Writing – review & editing. HL: Data curation, Writing – original draft.
