# Supplementary figures and images for: Protective effects of Lactococcus lactis subsp. lactis HFY14 supplementation on the brain, intestines, and motor function of antibiotic-treated mice
Source: Front Microbiol. 2024 Jun 14;15:1418556. doi: 10.3389/fmicb.2024.1418556 (PMC11211273; doi:10.3389/fmicb.2024.1418556)

## Slide 1
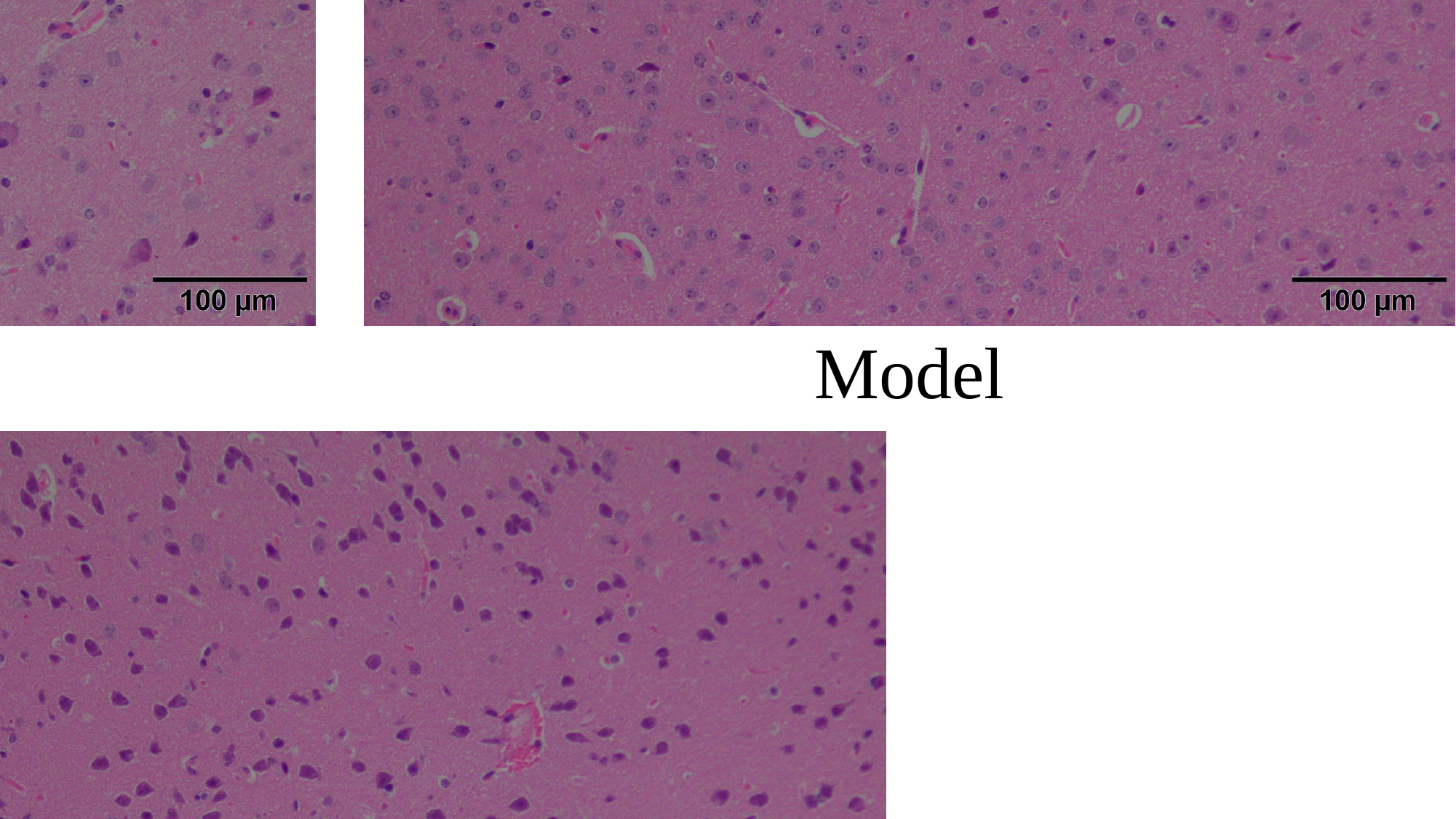

Model
Normal
CA
LLSLHFY14HD
LLSLHFY14LD

Supplement: Supplementary file 1 [file Data_Sheet_1.ZIP › DATA/Figure 3.pptx]

## Slide 1
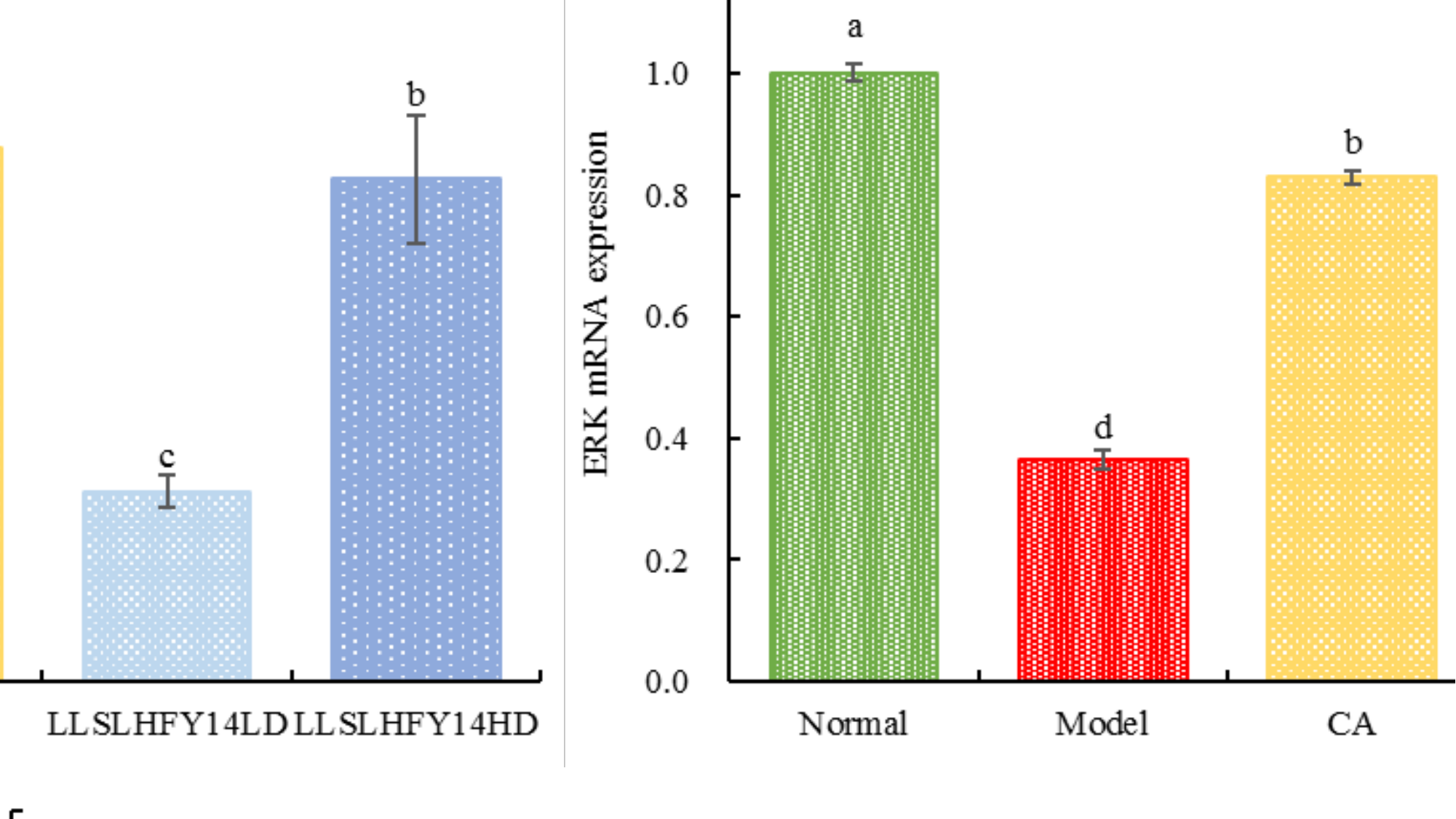

Supplement: Supplementary file 1 [file Data_Sheet_1.ZIP › DATA/Figure 4.pptx]

## Slide 1
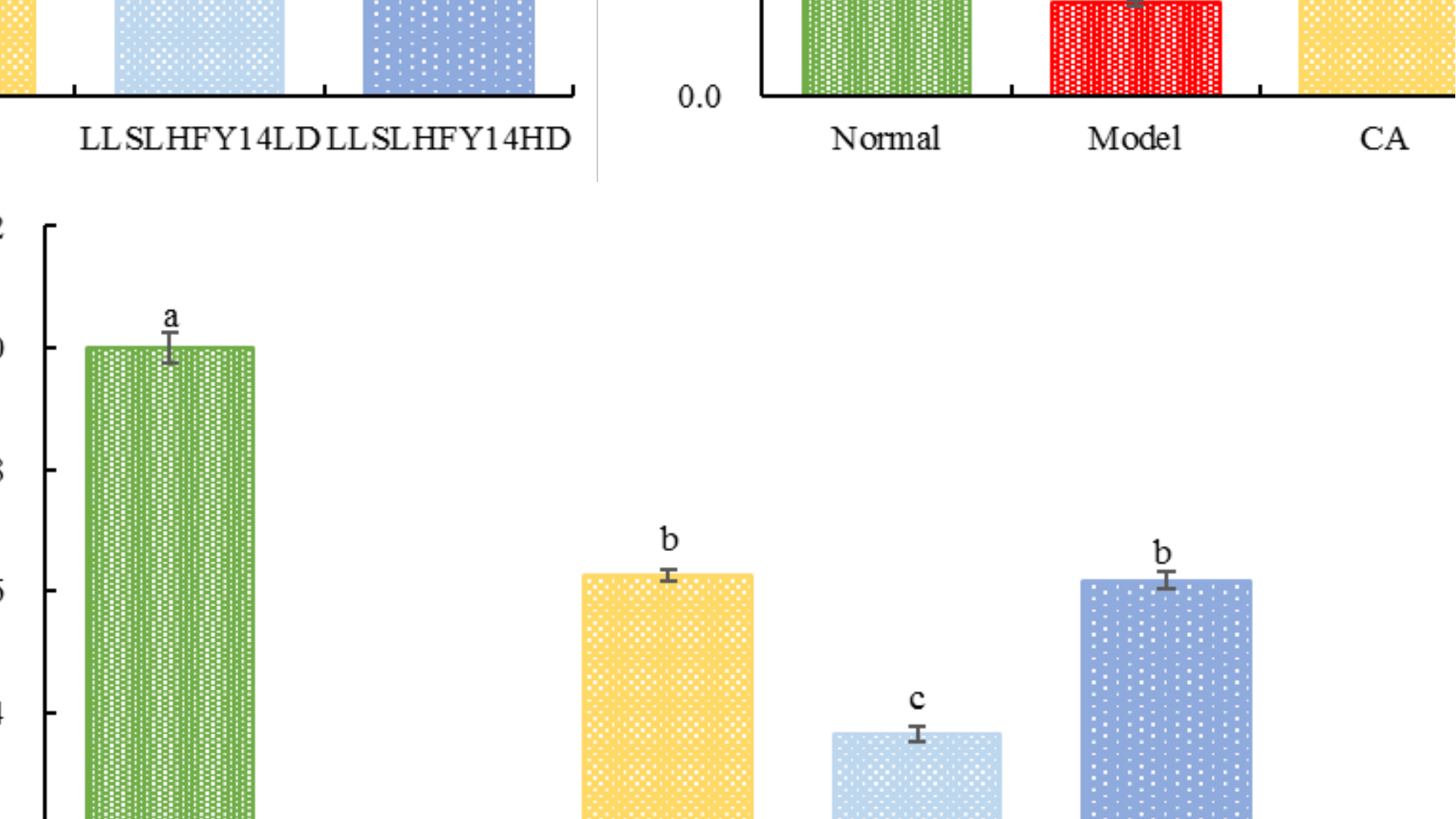

Supplement: Supplementary file 1 [file Data_Sheet_1.ZIP › DATA/Figure 5.pptx]

## Slide 1
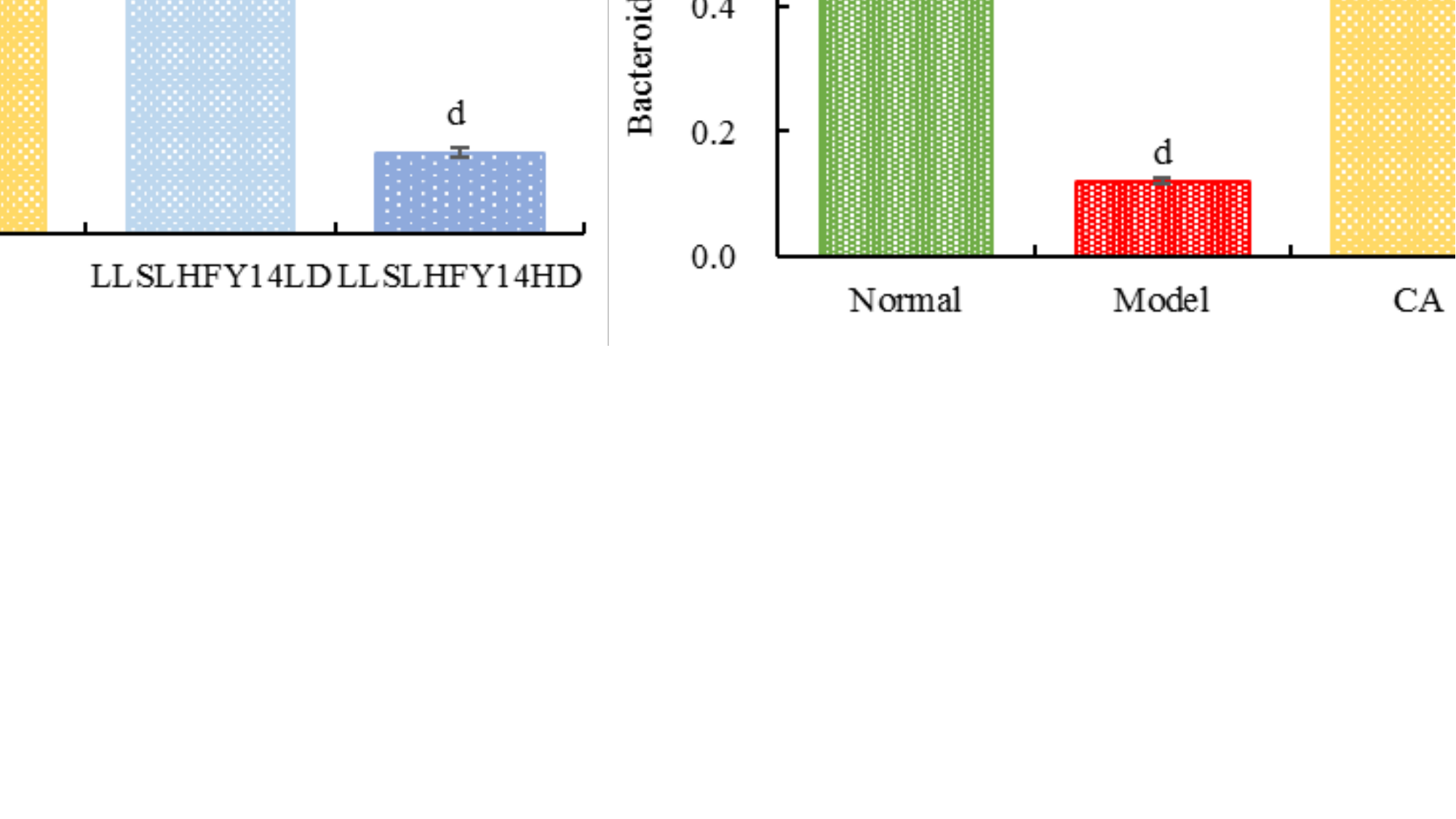

Supplement: Supplementary file 1 [file Data_Sheet_1.ZIP › DATA/Figure 6.pptx]
